# The role and regulation of Maf proteins in cancer

**DOI:** 10.1186/s40364-023-00457-w

**Published:** 2023-02-07

**Authors:** Yalan Deng, Liqing Lu, Huajun Zhang, Ying Fu, Ting Liu, Yongheng Chen

**Affiliations:** 1 Department of Oncology, NHC Key Laboratory of Cancer Proteomics, Laboratory of Structural Biology, Xiangya Hospital, Central South University, Changsha, 410008 Hunan China; 2 Department of Thoracic Surgery, Xiangya Hospital, Central South University, Changsha, 410008 Hunan China; 3 Department of Ultrasonic Imaging, Xiangya Hospital, Central South University, Changsha, 410008 Hunan China; 4 Department of Gastroenterology, Xiangya Hospital, Central South University, Changsha, 410008 Hunan China; 5 National Clinical Research Center for Geriatric Disorders, Xiangya Hospital, Central South University, Changsha, 410008 Hunan China

**Keywords:** Maf proteins, Proliferation, Metastasis, Biomarkers, Therapeutic targets

## Abstract

The Maf proteins (Mafs) belong to basic leucine zipper transcription factors and are members of the activator protein-1 (AP-1) superfamily. There are two subgroups of Mafs: large Mafs and small Mafs, which are involved in a wide range of biological processes, such as the cell cycle, proliferation, oxidative stress, and inflammation. Therefore, dysregulation of Mafs can affect cell fate and is closely associated with diverse diseases. Accumulating evidence has established both large and small Mafs as mediators of tumor development. In this review, we first briefly describe the structure and physiological functions of Mafs. Then we summarize the upstream regulatory mechanisms that control the expression and activity of Mafs. Furthermore, we discuss recent studies on the critical role of Mafs in cancer progression, including cancer proliferation, apoptosis, metastasis, tumor/stroma interaction and angiogenesis. We also review the clinical implications of Mafs, namely their potential possibilities and limitations as biomarkers and therapeutic targets in cancer.

## Background

The Mafs belong to basic leucine zipper transcription factors and are members of the AP-1 superfamily. The *v-Maf* was an oncogene that can cause musculoaponeurotic fibrosarcoma in vivo [1] and can transform chicken embryo fibroblasts in vitro, which was originally found in the genome of the avian transforming retrovirus AS42 [2]. The discovery of v-Maf led to the identification of its cellular counterpart c-Maf and related genes, which comprised the Mafs. Mafs are composed of two distinct subgroups classified according to their molecular size: large Mafs (approximately 240–340 amino acids), including c-Maf, MafB, MafA/L-Maf and neural retina-specific leucine zipper (NRL), and small Mafs (approximately 150–160 amino acids), including MafG, MafF and MafK [3]. They modulate the expression of a large number of genes, thereby regulating multiple cellular functions, including the cell cycle [4], proliferation [5], apoptosis [6], oxidative stress [7], inflammation [8], autophagy [9], drug resistance [10] and carcinogenesis [11]. The role they play in human health and illness is, therefore, of utmost importance. Deficiencies in Mafs function and dysregulation of Mafs are able to influence cell fate and contribute to tumor formation. There has been mounting evidence indicating that all Mafs mediate the initiation and progression of human cancers [12–14].

In this review, we conclude the molecular mechanisms regulating Mafs and how Mafs affect cancer development and progression. Our discussion also elucidates the potential of Mafs to be biomarkers during tumorigenesis. Finally, we discuss how to target Mafs for cancer therapy in upcoming research.

## Structures and physiological functions of Mafs

**Fig. 1:**
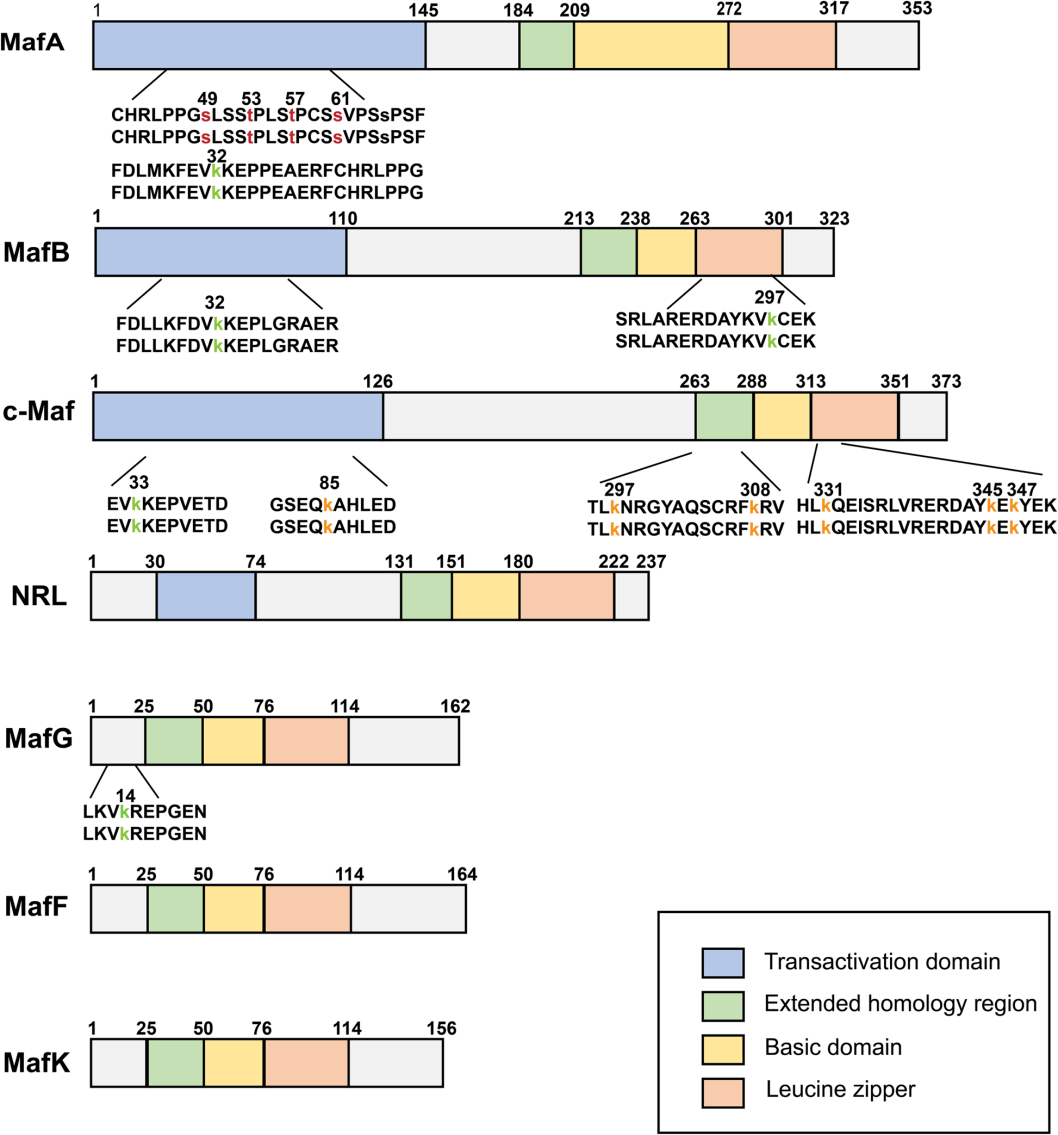
Schematic representation of human Mafs structures. The seven members of Mafs are shown with their sizes and domain arrangement [20, 25, 26]. Large Mafs contain a transactivation domain, an extended homology region, a basic domain, and a leucine zipper, while small Mafs lack a transactivation domain. The posttranslational modifications of Mafs in human cancer are indicated on the first line of amino acid codes below the bar, while the second row indicates highly conserved PTM sites in mouse. The red amino acid represents phosphorylation sites, the green indicates sumoylation sites and the yellow implies ubiquitylation sites

The basic region/leucine zipper (bZIP) domains of Mafs allow them to bind TPA responsive elements (TREs) or cAMP responsive elements (CREs) [15, 16]. Homodimers and heterodimers are formed through the leucine zipper domain, which is necessary to bind DNA. Mafs are characterized by the existence of a domain conserved among small Mafs and large Mafs called the extended homology region (EHR) that stabilizes DNA binding [17]. The palindromic sequence (TGCTGACTCAGCA) is a consensus binding motif for Mafs homodimers, which is also known as the Maf recognition element (MARE). This motif comprises a TGC flanking sequence contacted by the EHR domain and a TRE or CRE core region that is recognized by the basic domain. The basic region of Mafs contains a specific tyrosine residue (Tyr64), which is crucial to the recognition of GC boxes in MARE [18]. Notably, numerous natural target genes of Mafs possess only half of a MARE palindromic sequence. Nevertheless, if homodimers of Mafs are flanked by 5′-AT-abundant sequences, they are able to bind half of the MARE sites as well [19]. It is also possible that some target genes are regulated by Mafs containing heterodimers, with Mafs binding to half of each MARE site [12]. Since small Mafs lack a transactivation domain, homodimers within small Mafs or heterodimers formed with Cap-n-Collar (CNC) transcription factors are required to exert transcriptional repression or transactivation activity [20] (Fig. 1). Large Mafs have a highly conserved amino terminal domain that is associated with transactivation function [21]. The large Mafs are also able to activate transcription by enlisting the coactivators p300, CRE binding protein [22], P/CAF [23] and the TATA binding protein [24].

Mutations causing loss of function in mice implied that large Mafs participated in tissue specification before embryonic day E10 [27] and in terminal differentiation post E10 and during the postnatal period [28]. Loss-of-function of the small Mafs is related to megakaryocyte differentiation [29], neuronal homeostasis [30], the regulation of antioxidants [31] and embryo development [32] (Table 1). Within a different tissue, their roles seem to be specific [20, 33]. As Mafs perform multiple functions, the dysregulation of Mafs can cause a host of diseases, such as cancer.

**Table 1:** The physiological functions of Mafs

Subgroup	Mafs	Organ and/or cell type	Biological effect	Downstream	Cooperative factors	Reference
Large Mafs	c-Maf	Lens fiber cell	Lens formation and differentiation	Crystalline genes, *cyclin D1*	Prox-1, Pax6, CREB, SOX1, SOX2	[34, 35]
		LSECs, Foetal liver macrophages	embryological development of liver cells, LSECs specialization, immune tolerance	*F4/80*	MEIS2, GATA4	[36, 37]
		Kidney, proximal tubule cells, podocytes	functional differentiation	*GPx3*	Unknown	[37]
		T cell	T cell activation and differentiation	*IL-4, IL-10*	NFAT, CARMA1, IKKβ	[38]
		Pancreatic endocrine cells	α-cell differentiation, glucagon biosynthesis	Glucagon genes	Pax6	[39]
		Endochondral bone	Chondrocyte differentiation	*Col2a1*	SOX9	[40]
	MafA	Pancreatic β-cells	Insulin transcription and secretion	Insulin genes	PDX1, Neurog3	[41]
	MafB	Hindbrain	Segment formation in the hindbrain	*Hoxa3, Hoxb3*	KROX20	[27]
		Myeloid progenitors	Macrophage differentiation	Unknown	Unknown	[42]
		Kidney, podocytes	Formation of podocytes	Unknown	Unknown	[42]
	NRL	Retina	Differentiation of rod photoreceptors	*Rho*	CRX	[43]
Small Mafs	MafG	Hematopoietic system	Megakaryocyte differentiation, platelet release	*Bach2, Notch1*	MafK	[44, 45]
		Nervous system	Neurodevelopment	Unknown	Unknown	[44]
		Lens fiber cells	Lens formation	*Aldh3a1, Crygf, Hspb1, Pcbd1*	MafK	[46]
		Embryo	Embryo development	Unknown	MafF, MafK	[32]
	MafK	Hematopoietic system	megakaryocyte differentiation	Unknown	MafG	[47]
		Nervous system	Neurodevelopment	Unknown	MafG	[48]
	MafF	Hematopoietic system	Regulating oxidative stress	Unknown	MafG	[49]

*LSECs* Liver sinusoidal endothelial cells, *Rho* Rhodopsin, *Col2a1* Collagen type II ⍺1

## Upstream regulatory mechanisms of Mafs

**Fig. 2:**
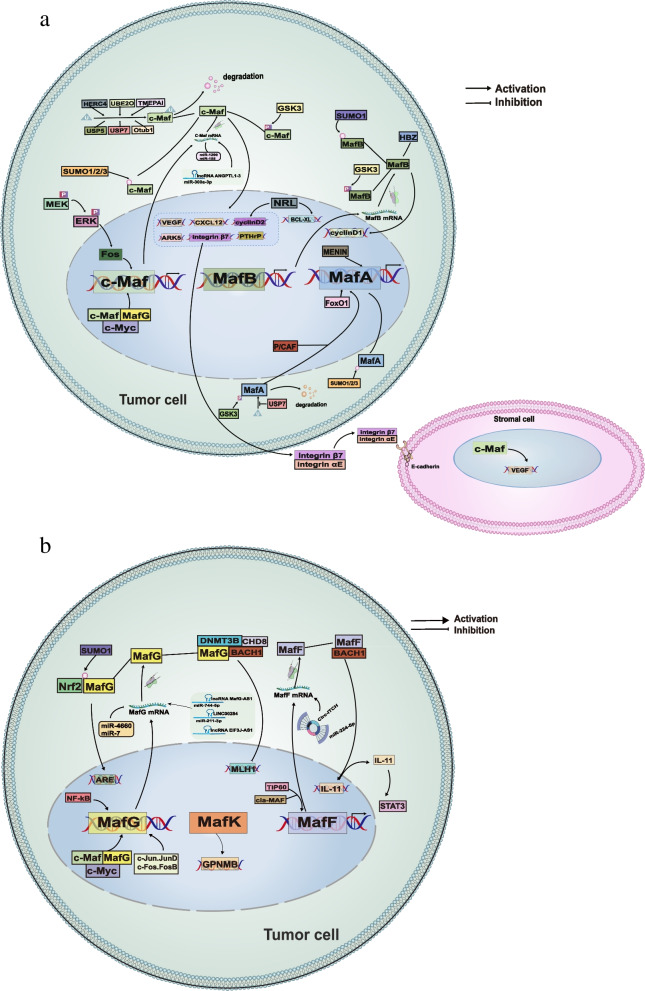
The regulatory network of Mafs in cancer. There are several different mechanisms involved in the regulation of large Mafs (**a**) and small Mafs (**b**) in tumor cells, including transcriptional regulation, noncoding RNAs, posttranslational modifications and protein–protein interactions. Large Mafs and small Mafs can influence tumor fate by regulating diverse downstream targets

Mafs activity is regulated in normal and cancer cells by multiple modes, including noncoding RNAs (ncRNAs), posttranslational modifications (PTMs) and protein–protein interactions (Fig. 2). We discuss various mechanisms for regulating Mafs both physiologically and pathologically, with a particular emphasis on their regulation during cancer development.

### Regulation of Mafs at the transcriptional level

Among the small Mafs, MafG is the downstream target of some transcription factors. A molecular study demonstrated that the MafG promoter contained AP-1, E-box, and NF-κB binding sites. The transcription factors p50, p65, AP-1 family (c-Jun, c-Fos, Fos-B, Fra-1, Fra-2) and c-myc are recognized as oncogenes due to their capacities to maintain malignant cell survival and offer signals for unlimited proliferation [50–52]. These transcription factors are highly expressed in esophageal cancer, prostate cancer and gastric cancer [53–55]. p50, p65, several AP-1 proteins (c-Jun, JunD, c-Fos and FosB) and c-myc can induce the promoter activity of MafG and transcriptionally activate the expression of MafG by directly binding to the NF-κB, AP-1 and E-box elements, respectively [56]. In addition to MafG, large Mafs can also be regulated by transcription factors. Raum et al. discovered a cis-regulatory region that was located approximately 8 kb upstream of the transcription start site in MafA. Pdx1, FoxA2 and Nkx2.2, all of which are crucial transcriptional regulators, can regulate MafA expression at the transcriptional level by binding to this cis-regulatory region [57]. In insulinoma cell lines, MENIN binds to MafA promoter sequences and transcriptionally regulates MafA protein and mRNA levels [58]. Kitamura et al. revealed that oxidative stress can control the transcription of MafA via FoxO1, which was a forkhead transcription factor participating in oxidative stress responses and metabolism [59]. Oxidative stress is a state of imbalance between reactive oxygen species (ROS) and antioxidants. Irreversible ROS damage to nucleic acids, lipids, and proteins may cause genetic and epigenetic alterations that drive the initiation of tumorigenesis [60]. Thus, MafA may take part in cancer progression based on the regulation of redox signaling, in which the underlying molecular mechanism needs to be further studied. Electrophoresis mobility shift experiments and DNase I foot-printing analysis demonstrated that the 5'-flanking and 5'-noncoding regions of the rat c-Maf gene harbored at least three Pax6-binding sites, which robustly activated the c-Maf promoter construct [61]. The FOS, downstream of MEK1 and ERK kinase, binds to the c-Maf promoter and directly activates c-Maf expression in human myeloma cells [62]. Huang K et al. demonstrated that MyoD activated the mouse MafB promoter by co-transfection analysis [63]. Although Mafs have similar structures and promoter regions (slightly different between large Mafs and small Mafs) [61], the upstream factors regulating each member of Mafs expression are different. Under such intricate and finely detailed regulation, each member specifically participates in diverse signaling pathways to precisely modulate tissue development or influence cancer progression.

### ncRNAs regulate Mafs at the posttranscriptional level

ncRNAs are unable to be translated into proteins, but can regulate gene expression at both the transcriptional and posttranscriptional levels in many cellular processes [64–66]. There are several major types of ncRNAs: small RNAs including miRNAs [67], circular RNAs (circRNAs) [68] and long noncoding RNAs (lncRNAs) [69] et al. Recently, it was documented that ncRNAs regulated tumor growth through targeting transcription factors, including Mafs (Table 2).

**Table 2:** ncRNAs targeting Mafs in cancer

Cancer types	ncRNAs	Mafs	Functions of the interaction	Reference
Osteosarcoma	miR-4660	MafG	Inhibits Osteosarcoma cell growth	[70]
Lung adenocarcinoma	lncRNA MAFG-AS1	MafG	Suppresses the proliferation and induce apoptosis of lung adenocarcinoma cells	[71]
	miR-7	MafG	Restores resistance to cisplatin	[72]
Prostate cancer	lncRNA EIF3J-AS1	MafG	Promotes proliferation and metastatic ability of prostate cancer cells	[73]
Oral cancer	LINC00284	MafG	Promotes cell proliferation and migration of oral squamous cell carcinoma	[74]
Laryngocarcinoma	miR-1290	c-Maf	Suppresses apoptosis of laryngeal squamous cell carcinoma cells	[75]
Neurofibroma	miR-155	c-Maf	Promotes tumor growth in plexiform neurofibroma	[76]
Multiple myeloma	lncRNA ANGPTL1-3	c-Maf	Increases bortezomib sensitivity of multiple myeloma cells	[10]
Nasopharyngeal carcinoma	miR-223	MafB	Inhibits cell proliferation and migration of nasopharyngeal carcinoma	[77]
Liver cancer	circ-ITCH	MafF	Inhibits the proliferation and induces cell apoptosis of hepatocellular carcinoma	[78]
	cia-MAF	MafF	Impairs tumorigenesis, self-renewal and metastatic capacities in liver cancer	[79]

miRNA consists of 18–25 nucleotides and regulates the translation of mRNAs that perform multiple biological functions, such as cell proliferation, differentiation, and homeostasis maintenance [80–83]. They bind to the mRNA 3'-untranslated region (3'UTR) to inhibit translation via forming an RNA-induced silencing complex (RISC) with other cofactors [84–87]. miR-4660 and miR-7 that directly contact the 3’ untranslated region of MafG mRNA suppress osteosarcoma cell growth and chemosensitivity to cisplatin in non-small-cell lung cancer (NSCLC) respectively [70, 72]. Moreover, c-Maf is a target of miR-1290 and miR-155 in laryngeal squamous cell carcinoma and plexiform neurofibromas. miR-1290 and miR-155 reduce both the mRNA level and protein expression of c-Maf by directly interacting with the 3’UTR of c-Maf [75, 76]. In addition, miR-223 exerts a suppressive effect on nasopharyngeal carcinoma via targeting MafB and reducing MafB expression [77].

CircRNAs are circular RNA molecules with a continuous and covalent closed loop, thus increasing their stability compared to linear RNA molecules [88]. CircRNAs are defined as miRNA sponges to control target gene expression [89–92]. In recent studies, it was demonstrated that circRNAs regulate the expression of Mafs. For instance, enforced expression of circ-ITCH inhibited the proliferation and induced apoptosis of hepatocellular carcinoma cells via upregulating MafF expression by acting as a miR-224-5p sponge [78]. However, circular RNA cia-MAF recruited the TIP60 complex to the MafF promoter region and, as a result, contributed to MafF expression. Loss of cia-MAF blocked the interaction between the TIP60 complex and the MafF promoter and resulted in impaired liver tumorigenesis, self-renewal and metastatic abilities [79]. The different functions of MafF in liver cancer might result from a complicated regulatory network of circRNAs.

LncRNAs refer to noncoding RNAs greater than 200 nucleotides in length [69]. An increasing number of studies have shown that certain lncRNAs participate in cancer progression through sponging miRNAs [93]. The Mafs expression can be modulated by lncRNAs as well. The lncRNAs MAFG-AS1, lncRNA EIF3J-AS1 and LINC00284 upregulate MafG to promote proliferation and metastasis and inhibit apoptosis by sponging miR-744-5p and miR-211-3p in lung adenocarcinoma, prostate cancer and oral squamous cell carcinoma [71, 73, 74]. Likewise, lncRNA ANGPTL1-3 decreases the bortezomib sensitivity of multiple myeloma cells through retaining miR-30a–3p and restraining its binding to c-Maf [10]. In conclusion, it appears that tumor cells activate or inhibit Mafs through various ncRNA-based mechanisms to sustain malignancy. The ncRNAs are very stable in biological fluid due to their localization in exosomes [94]. Detecting ncRNAs may be a convenient and indirect approach to evaluate the expression and activity of Mafs. Thus, deep understanding of the relationship between ncRNAs and Mafs is crucial for developing Mafs-based strategies.

### The PTMs contribute to the regulation of Mafs

Posttranslational modifications (PTMs) are known for their key roles in modulating the functions of proteins, which influence their degradation, transcriptional activity, protein–protein interactions and subcellular location [95]. The Mafs were documented to undergo several PTMs, such as phosphorylation and ubiquitylation, that altered their function to affect their target gene expression [23, 96].

In vivo, the primary MafA kinase is glycogen synthase kinase 3 (GSK3), which unceasingly phosphorylates MafA on serine (S)49, threonine (T)53, threonine (T)57, and serine (S)61, implying that a priming kinase unknown to us phosphorylates serine (S)65 [23, 97]. Similar phosphorylation sites are present in c-Maf and MafB and both proteins are also phosphorylated by GSK3 [23], providing that these phosphorylation sites are highly conserved in large Maf proteins. In the case of MafA, GSK3-mediated phosphorylation pairs two antergic processes: its degradation by ubiquitylation and increased transactivation activity by boosting recruitment of the coactivator P/CAF [23, 97]. The role of MafA in cell differentiation as well as oncogenesis can be controlled by phosphorylation [23]. Furthermore, MafB and c-Maf can be ubiquitinated and subsequently degraded in proteasomes by the ubiquitin-conjugating enzyme UBE2O, the ubiquitin ligase HERC4 and NEDD4, while stabilized by the deubiquitinating enzymes USP5, USP7 and Otub1 [11, 98–102]. It is possible that the process of ubiquitylation contributes to the abort of transcriptional responses or, in some circumstances, may function to prevent inappropriate Mafs expression.

Sumoylation is common in the regulation of Mafs. The sumoylation of MafB on lysine (K)32 and lysine (K)297 causes the repression of its transactivation activities and its ability to terminate the cell cycle and to promote colorectal cancer tumorigenesis [103]. MafA is also posttranslationally modified by SUMO proteins at a conserved lysine residue in the amino-terminal transactivation domain, which negatively regulates its transcriptional and oncogenic activities [104]. c-Maf colocalizes with two SUMO ligases in the nucleus, which sumoylate c-Maf at lysine 33 and attenuate its transcriptional activity [105]. In addition, sumoylation modulates the functions of small Mafs by altering their interaction with other proteins. For instance, SUMO-1-mediated sumoylation is required for MafG/Nrf2 heterodimer formation and antioxidant response element binding, which can protect hepatocytes from oxidative stress injury and liver cancer development [106]. Depending on posttranslational modifications, Mafs functions will synergize or compete with one another. Thus, further studies on the PTMs of Mafs are necessary to gain a deeper understanding of Mafs molecular functions and regulatory mechanisms.

### The role of protein–protein interactions in modulating Mafs activity

Interactions between proteins can alter the activity of Mafs. Known as transcription factors, Mafs stimulate or inhibit target gene expression by interacting with various proteins. This means that Mafs activity depends on the expression of coregulators and general transcription factors in a specific cell type. Yang et al. revealed that MafG, c-Maf, c-Myc and MATα1 interacted with one another directly in cholangiocarcinoma (CCA). The promoter regions of these genes have E-boxes that are bound by MATα1 in normal liver and switched to c-Myc, c-Maf and MafG in CCA cells. The E-box positively regulates c-Myc, c-Maf and MafG [107]. Furthermore, Hox proteins, including Hoxd12, MHox, Prx1, Phox1 and Pmx1, can interact with Mafs (c-Maf, MafB, MafK, MafF, and MafG) through the homeodomain of Hox proteins and the bZIP domain of Mafs. The co-expression of Hox proteins inhibited the transactivation and transforming activity of Mafs, which implied that the interaction of a set of Hox proteins with Mafs may disturb not only their oncogenicity but also their physiological roles [108]. In addition, HTLV-1 basic leucine-zipper (bZIP) factor (HBZ), which is closely associated with adult T-cell leukemia, interacts and heterodimerizes with MafB via each bZIP domain. HBZ abrogates the Maf recognition element (MARE) binding activity of MafB and reduces steady-state MafB levels [109]. Therefore, interactive partners also play an indispensable role in the target gene expression of large Mafs in the presence of the transactivation domain.

Mafs modulate a number of physiological processes and exhibit a characteristic manner in which transcription factors integrate signaling in response to environment incentive. As with other oncoproteins, their dysregulation can contribute to tumorigenesis.

## Importance of Mafs in cancer development

**Fig. 3:**
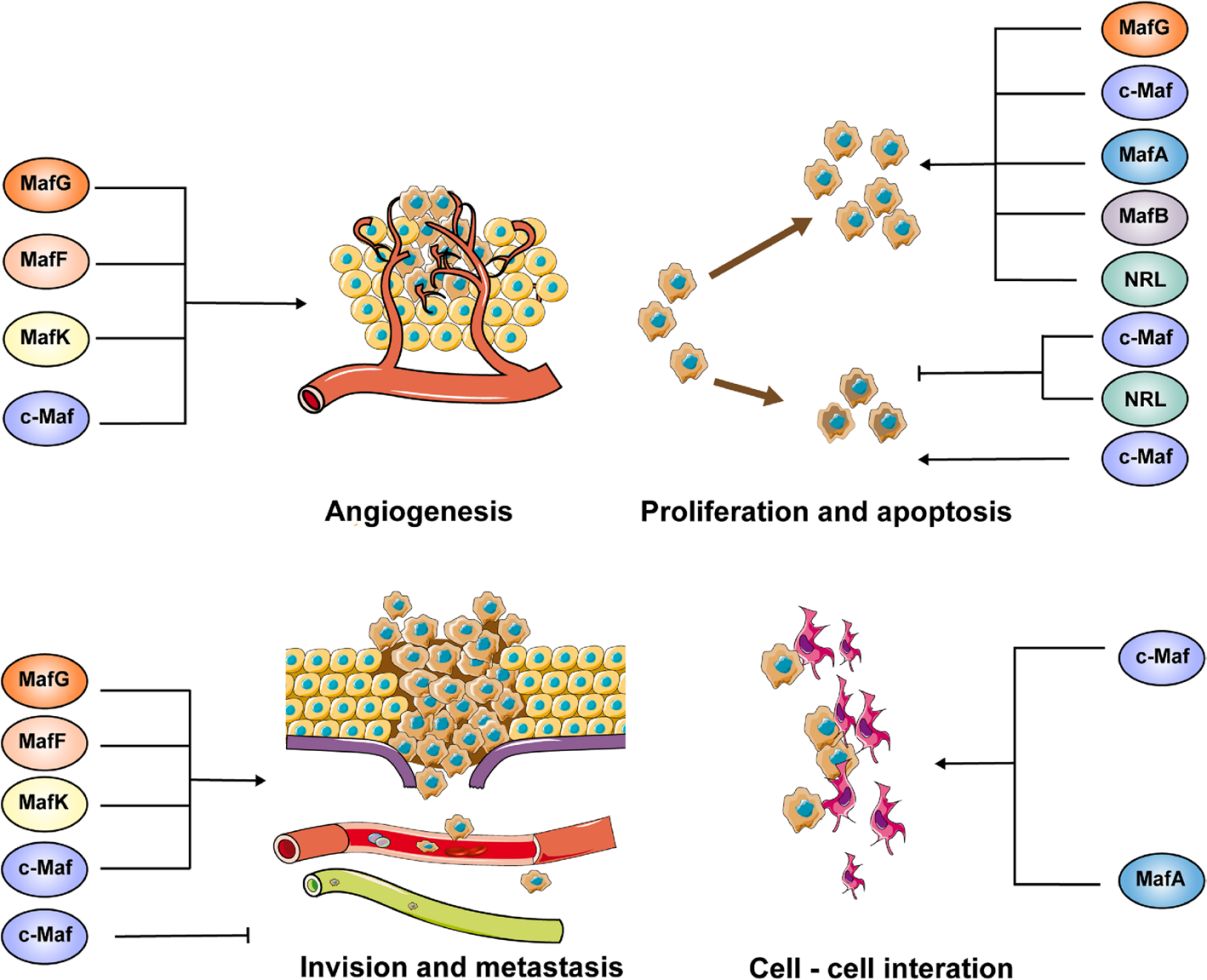
Role of Mafs in cancer progression. The four large and three small Mafs could function as oncoproteins, and they facilitate cancer progression through promoting proliferation, invasion, metastasis, EMT, angiogenesis, and tumor-stroma interactions and inhibiting apoptosis in a variety of cancers. However, in laryngeal squamous cell carcinoma and MPNST, c-Maf works as a tumor suppressor through regulating apoptosis and metastasis

The strictly regulated activity and expression of Mafs create a dynamic, balanced transcriptional network that is essential for optimal cell function and tissue formation [13, 110]. Thus, dysregulation of Mafs has a direct impact on proliferation, differentiation, apoptosis, migration and invasion, which is closely tied to tumor formation, progression, metastasis, angiogenesis, tumor/stroma interaction and drug resistance (Table 3, Fig. 2, and 3). It will be possible to develop better therapies and diagnostic approaches for cancer with a deeper understanding of Mafs function in cancer development.

**Table 3:** Functional roles of Mafs pathway in cancer

Subgroup	Mafs	Cancer types	Key roles	Reference
Large Mafs	c-Maf	Multiple myeloma (MM)	c-Maf subgroup of MM is characterized by high proliferative index and is associated with cyclin D2 overexpression	[111]
			Stabilized by USP7 and USP5 and exerts anti-apoptosis effect on myeloma cells	[11, 112]
			Influences MM invasion process through regulating CXCL12 and ARK5	[113, 114]
			Increases the interaction between tumor cells and stroma by increasing the expression of integrin β7 in MM	[115]
			Induces VEGF expression and promotes marrow neo-angiogenesis	[116]
		Lung cancer	Berbamine inhibited the migration and invasion abilities of non-small-cell lung cancer cells by downregulating c-Maf	[5]
			Facilitates tumor-associated macrophages polarization and promotes angiogenesis in NSCLC	[117]
		Breast cancer	Promotes breast cancer bone metastasis and may act as a biomarker of bone relapse	[118]
	MafA	MM	Stabilized by deubiquitinating enzyme USP7 and suppresses myeloma cell apoptosis	[11]
	MafB	Colorectal cancer	Promotes colorectal cancer cell proliferation via regulating cell cycle and is correlated with advanced TNM stage	[103]
		Liver cancer	Promotes HCC proliferation through enhancing Cyclin D1	[119]
		Osteosarcoma	Drives cancer stemness in osteosarcoma. High MafB expression is strongly correlated with poor prognosis	[120]
		Ovarian cancer	Promotes the proliferation and invasion of ovarian cancer cells and reduces olaparib/cisplatin sensitivity	[121]
	NRL	Medulloblastoma	Protects cells from apoptosis and mediates cell cycle progression	[122]
Small Mafs	MafG	Colorectal cancer	Differentially expressed between highly metastatic colorectal cancer and nonmetastatic colorectal cancer	[123]
			Enhances proliferation by heterodimerizing with Bach1 and recruiting CHD8 and DNMT3B	[124]
		Liver cancer	Promotes proliferation through heterodimerizing with Nrf2 or interacting with c-Myc and c-Maf	[56, 125]
		Lung cancer	Accelerates cell proliferation and inhibits cell apoptosis in lung adenocarcinoma	[71]
	MafK	Breast cancer	Induces EMT and promotes tumor invasion in vivo	[126]
	MafF	Liver cancer	Drives liver tumor-initiating cells metastasis and antagonizes the retinoid-mediated suppression of HCC invasion	[79, 127]
		Breast cancer	Promotes tumor invasion through heterodimerizing with Bach1 and activating IL11/STAT3 pathway	[128]

### Implications of Mafs in cancer proliferation and apoptosis

In both physiological and pathological circumstances, oncogenes can regulate cell proliferation. Mafs not only participate in the process of cell proliferation but are also responsible for blocking the progression of the cell cycle during development. For example, Mafs are able to boost the expression of the cyclin-dependent kinase inhibitor p27 [96, 129].

Cancer is characterized by constant proliferative capacity and resistance to apoptosis of malignant cells. Initially, the dominant contributions to the understanding of how Mafs regulate cancer proliferation and initiation originated from studies on multiple myeloma (MM). Seven frequently occurring primary translocations are commonly believed to initiate oncogenic processes of MM, in which the immunoglobulin heavy chain (IgH) locus is involved [130, 131]. Among these translocations, three concern large Mafs [132]. It was first discovered in 1990 that a recurrent t (14;16) (q32.3; q23) translocation caused overexpression of c-Maf due to juxtaposition with enhancers of the IgH locus [133]. A similar translocation involving MafB [134, 135] and MafA [132] was described shortly afterwards in recent years. Translocations affecting large Mafs were seen in approximately 9% of MM cases, with c-Maf translocations accounting for 6%, MafB for 3%, and MafA for less than 1% [132], which were tied to various losses on chromosome 13 [136]. Translocations of MafB occurred most frequently at the beginning of MM development, while the prevalence of c-Maf translocations was more common as the disease progresses [111, 132, 136, 137]. It is an appropriate explanation of this observation that MafB has weak transforming activity in cell culture [138, 139], and there is a possibility that the pathological outcomes of various large Mafs translocations are not identical. Paradoxically, it has been found that the proliferation of both primary fibroblast cultures [139] and MM cell lines [115] can be induced by oncogenic Mafs. Cyclin D2 is involved in controlling the cell cycle entry and proliferation stage. The c-Maf subtype of MM exhibits high proliferative markers and is tightly linked to *cyclin D2*, a gene target of c-Maf [111, 115]. In angioimmunoblastic T-cell lymphomas that express c-Maf and in T-cell lymphomas of transgenic mice with overexpressed c-Maf in the lymphoid compartment, deregulated cyclin D2 expression has been shown as well [140, 141]. The oncogenic role of large Mafs in inducing cell proliferation has also been verified in other cancers, including prostate, lung, colorectal, nasopharyngeal, oral and liver cancer as well as plexiform neurofibroma and glioma [5, 56, 103]. For example, knockdown of MafB attenuated colorectal cancer cell proliferation via arresting the cell cycle at G0/G1 phase in vitro [103]. Moreover, *cyclin D1*, another important regulator of cell cycle progression, is identified as a direct target gene of MafB in hepatocellular carcinoma. Enforced overexpression of MafB promotes hepatocellular carcinoma proliferation by enhancing cyclin D1 [119]. Similar observations have been made in ovarian cancer [121] and osteosarcoma [120]. It is previously reckoned that NRL has not been implicated in human cancer, and it is solely viewed as a terminal differentiation factor of rod photoreceptors [33]. However, Garancher A et al. demonstrated that NRL could bind to the promoter region of cyclin D2 and mediate cell cycle progression through controlling cyclin D2 expression [122]. Cyclin–CDK complexes are also likely to be regulated by large Mafs in primary fibroblast cultures, causing the acceleration of the cell proliferation rate. However, it does not appear that these proteins were directly involved with cyclin D2 [139]. Diverse mechanisms of disruption of the cell cycle are a feature of Mafs-mediated transformation.

In addition to cell cycle progression, large Mafs-induced cell proliferation also results from anti-apoptotic activity. Cancer cells are able to acquire resistance to apoptosis and a higher survival rate by constitutively activating the expression of large Mafs through diverse mechanisms, including large Mafs translocation; disruption of large Mafs ubiquitylation by upregulating the deubiquitinating enzyme Otub1, ubiquitin-specific protease USP5 or USP7; Ras kinase cascade mediated induction of large Mafs [11, 62, 112, 142]. Moreover, cancer cells induce c-Maf expression in response to chemotherapeutic exposure, rendering them intrinsically resistant to apoptosis [143]. NRL directly protects cancer cells from apoptosis by transcriptionally inducing BCL-XL expression [122]. Thus, NRL activation might explain the high BCL-XL levels that occur in the absence of translocations, amplifications, or other known epigenetic changes in the *BCL-XL* loci [144]. *p53* is a well-known tumor suppressor gene, and the accumulation of p53 can cause cancer cell apoptosis [145]. p53 negatively regulates c-Maf expression through indirectly enabling the induction of miRNAs [146]. Notably, the c-Maf activates MARE-mediated p53 expression, suggesting a negative feedback regulatory mechanism between c-Maf and p53 [147]. In addition to inhibiting the degradation of c-Maf in proteosomes, USP7 can stabilize MDM2, a ubiquitin ligase of p53, reducing p53 expression and promoting cancer cell survival [148]. Accordingly, the upstream regulation of oncoproteins or tumor suppressors determines their expression level and cancer cell fate. However, a recent study discovers that large Mafs might have a positive effect on apoptosis. Loss or mis-localization of c-Maf nuclear expression is observed in laryngeal squamous cell carcinoma and genes involved in the regulation of apoptosis harbor the c-Maf binding motif in their promoter region, which implies that c-Maf acts as a tumor suppressor by regulating apoptosis [75]. Hence, in order to determine whether Mafs act as suppressor or oncogenes, further research is needed in a wider variety of cancer types to optimize the use of Mafs-based therapeutics in specific types of cancers.

Since lacking transactivation domain, small Mafs participate in tumor progression through forming heterodimers with the CNC family proteins p45 (large nuclear factor erythroid 2 (NF-E2) subunit), Nrf1 (NF-E2-related factor 1), Nrf2, and Nrf3, as well as Bach1 and Bach2 [149]. The Keap1/Nrf2/small Mafs pathway is a major sensor for the cellular response to stress [150]. Under normal conditions, Nrf2 is sequestered by the Cul3-Keap1 complex in the cytoplasm and targeted for ubiquitination and proteasomal degradation. Upon exposure to oxidative stresses and electrophilicity, keap1-Nrf2 complex activity is disrupted, leading to the translocation of Nrf2 to the nucleus and activation of target genes by small Mafs/Nrf2 heterodimers. The heterodimers bind to the antioxidant response element (ARE) located in the regulatory region of these target genes and confer advantages with respect to stress resistance and cell proliferation in tumor cells [151]. Oncogenic proteins that promote proliferation, including KRASG12D, BRAFV619E, and MYC, also induce the transcription of Nrf2 and activate the small Mafs/Nrf2 antioxidant program in pancreatic cancer [152]. The B-Raf protooncogene variant BRAF (V600E) upregulates MafG, which heterodimerizes with Bach1 and recruits both chromodomain helicase DNA-binding protein 8 (CHD8, a chromatin remodeling factor) and the DNA methyltransferase DNMT3B, leading to transcriptional silencing of tumor suppressor genes and cancer proliferation [124]. In addition, small Mafs form a complex with methionine adenosyl transferase α1 (MATα1), c-Myc and c-Maf to regulate tumor growth in CCA independent of CNC family proteins [107]. MATα1 takes part in the synthesis of the biological methyl donor SAMe, which blocks the effect of mitogens on ERK and AKT signaling in several cancers [153]. It is an emerging area in small Mafs research that will reveal the interplay of the cellular detoxification program and PI3K-AKT signaling pathway.

### Implications of Mafs in cancer invasion and metastasis

Metastasis refers to a complicated multistage pathological process that significantly contributes to cancer morbidity and mortality, in which a number of distinct steps are involved, including the destruction of the basement membrane, undergoing epithelial-to-mesenchymal transition (EMT), remodeling of the extracellular matrix (ECM) in the stroma, overcoming anoikis, and invading and reinitiating of malignant cells at the secondary sites [154]. As malignant cells invade and metastasize, they colonize secondary locations that are far from the primary tumor.

c-Maf is one of the large Mafs that is most closely associated with tumor invasion and metastasis. c-Maf is differentially expressed in circulating tumor cells originating from the bone-only metastasis group and the extraskeletal metastasis group [155]. Pharmacological inhibition of c-Maf suppresses the migration and invasion abilities of non-small-cell lung cancer cells through unknown mechanisms [5]. Conversely, 16q23 gain or c-Maf overexpression promotes breast cancer bone metastasis, a process that may be mediated by downregulation of the c-Maf target gene PTHrP, a crucial predictor for bone relapse in advanced breast tumors [118, 156]. To clear a path for migration, cancer cells secrete extracellular matrix remodeling enzymes, such as matrix metalloproteinase 13 (MMP13), which also liberate growth factors and cytokines trapped in the ECM [157]. c-Maf activation enhances MMP13 promoter activity via binding the AP-1 element and has been identified as an important transcriptional regulator of MMP13 [158]. An important hallmark in the c-Maf subtype of MM is the frequent occurrence of genes that trigger invasive process, like *ARK5* and *CXCL12* [113, 114, 159]. *ARK5* encodes a serine(S)/threonine(T) kinase that belongs to the AMP-activated protein kinase family and is also activated downstream of Akt. Previous studies have demonstrated that ARK5 participates in the process of invasion and metastasis [160], and the phenotype of increased invasiveness has been observed in MM cell lines overexpressing ARK5 [114]. The expression of ARK5, dysregulated in ARK5-driven T-cell lymphomas in transgenic mice as well as AITLs in humans, correlates well with Maf-transforming activity [140]. Furthermore, in primary fibroblasts, GSK3-mediated phosphorylation of c-Maf deregulates the expression of genes linked with extracellular matrix remodeling and cell invasiveness [23]. Another study indicates that c-Maf has anti-metastatic properties. Migratory and circulating metastatic cells have to overcome anoikis, the cell death process initiated when a cell loses contact with the ECM for a prolonged period of time [161]. The effect of c-Maf on anoikis dictates this opposite phenotype. c-Maf reduces the anchorage-independent growth and metastasis ability of malignant peripheral nerve sheath tumor (MPNST) through targeting DEPTOR, a negative regulator of the AKT/mTOR pathway [162].

Three small Mafs have also been implicated in regulating cancer metastasis. During EMT, epithelial cells lose expression of the adhesion protein E-cadherin in favor of N-cadherin. In cancer cell lines, MafK promotes EMT by downregulating E-cadherin expression through targeting transmembrane glycoprotein non-metastatic B (*GPNMB*), which is a potent inducer of EMT [126, 163]. Dysregulation of MafG has been identified by proteomic profiling in highly metastatic cancer cells relative to nonmetastatic cancer cells [123]. Liver tumor-initiating cells (TICs), which have the ability to self-renew, differentiate, and produce new tumors, are involved in liver tumorigenesis and metastasis [164]. Circular RNA cia-MAF overexpression drives liver TIC metastasis by recruiting the TIP60 complex to the MAFF promoter and promoting MAFF expression [79]. CNC proteins, especially Bach1, are indispensable partners for small Mafs to influence the process of cancer metastasis and invasion. The master regulatory role of Bach1 in cancer metastasis has been well discussed in many excellent reviews [165–167]. Bach1 is stabilized by antioxidants, and this effect is driven by reduced levels of ROS and free heme [168]. Heterodimers of small Mafs and Nrf2 stimulate cancer metastasis by inducing HO-1, the enzyme catabolizing heme, leading to elevated antioxidants and Bach1 stabilization [169]. On the other hand, small Mafs directly dimerize with Bach1 to transcriptionally activate MMP1, another ECM remodeling enzyme, and IL11, a cytokine involved in osteolysis-mediated bone metastasis, resulting in an increase in bone metastasis [128, 165]. Small Mafs/Nrf2 and small Mafs/Bach1 can compete for MARE/ARE binding. Therefore, the activation of distinct downstream genes is highly dependent on the quantity and abundance of small Mafs binding partners.

### Implications of Mafs in the interaction between the tumor and the stroma

As a crucial component of the tumor microenvironment, the most striking characteristics of the tumor stroma are its ability to actively promote cancer growth, angiogenesis, invasion and migration, immune escape, and resistance to cancer treatment [170, 171]. The function of stromal cells and their interplay with cancer cells within the tumor environment are controlled by the expression and secretion of many pivotal signaling molecules, including growth factors [172–174], chemokines [175–177], cytokines [178–181], and proteolytic enzymes [182–184]. During tumor development, the intimate tumor/stroma interaction influences the tumor microenvironment and determines the fate of malignant cells. The exploration of the underlying mechanisms governing this complex and dynamic interaction plays a key role in cancer diagnosis and therapy.

c-Maf enhances the tumor cell-stroma interaction during oncogenesis [115, 185] through stimulating the expression of *integrin β7* [115], which is a c-Maf transcriptional target [186]. Integrin β7 works along with the heterodimeric partner integrin αE to facilitate the attachment of cancer cells to bone marrow stromal cells, the surface of which expresses the integrin-binding protein E-cadherin. As a result of the interaction between cancer cells and stromal cells, the expression of vascular endothelial growth factor (VEGF) is enforced, helping to modulate a tumor-adapted microenvironment [115]. The expression of integrin β8 [23] is also dysregulated in large Maf-transformed cells, suggesting that Mafs might contribute to oncogenesis by disrupting the integrin signaling pathway. Intriguingly, the properties of large Mafs to mediate cell–cell interactions may be essential for their critical physiological roles as well. Defective cell-to-cell interactions in specific organs are observed by knocking out different large Mafs [41, 187]. In MafA-knockout mice, the pancreatic islets exhibit an unorganized architecture, which is featured with inappropriate intermixing of diverse cell types [41]. The *traffic-jam*, the D. melanogaster ortholog of the large Maf gene, is exclusively expressed in somatic gonadal cells directly adjacent to germline cells. The *traffic jam* dysfunction leads to improper intermingling of somatic cells with germ cells, resulting in defective differentiation of germ cells. The dysregulation of adhesion molecules, such as DE-cadherin, the E-cadherin ortholog from D. melanogaster, is implicated in this pathological process [187]. However, evidence that Mafs regulate the interaction between the stroma and tumor in other types of cancer has not been found and needs to be further explored. Therefore, under both physiological and pathological processes, c-Maf and MafA are crucial mediators of stroma/tumor interactions**.** These discoveries on Mafs and their target genes give rise to broad interest, as they identify a category of oncogenes that facilitate mutual interplay between the tumor and stroma, instead of enabling tumors to provide self-proliferative signals.

### Implications of Mafs in cancer angiogenesis

The growth of solid tumors is restrained by the availability of oxygen and nutrients. The hypoxic microenvironment of tumors activates the transcription factor HIF-1α, which initiates a signaling cascade that activates the transcription of growth factors (such as the VEGF family), cytokines and ECM remodelers to form the vasculature [188]. Constant and aberrant angiogenesis is indispensable for malignant processes, such as tumor growth and metastatic spread, which are key players in tumorigenesis [189]. In MM, c-Maf induces VEGF expression that is produced both by myeloma and marrow stroma cells, which promotes bone marrow neo-angiogenesis [116]. Tumor-associated macrophages (TAMs) reside in hypoxic area of tumors and serve as angiogenesis-promoting cells by producing pro-angiogenic factors and MMPs [190]. c-Maf is an essential controller of TAM polarization and recruitment. And c-Maf induces VEGFA expression in TAMs, which is a member of the VEGF family and regulates angiogenic sprouting [117]. The cell surface peptidase CD13/APN is expressed by activated endothelial cells in tumor vessels in response to angiogenic growth factors. CD13 inhibition reduces angiogenesis and halts tumor growth in vivo [191]. c-Maf occupies a crucial regulatory region of the CD13 proximal promoter and substantially activates the transcription of CD13 [192]. Therefore, c-Maf not only influences tumor cells but also affects various cell types in tumor microenvironment to expedite the process of vasculogenesis. Three small Mafs (MafK, MafF, and MafG) have also been identified as master regulators central to VEGFA signaling, and these small Mafs are upregulated by VEGFA, implying the significant role of small Mafs in angiogenesis [193]. Moreover, the small Mafs/Nrf2 heterodimer target gene quinone oxidoreductase-1(*NQO1*) encodes a protein that directly interacts with HIF-1α and prevents its degradation, which consequently promotes the expression of the VEGF family and angiopoietin [194, 195]. The small Mafs/Nrf2 might also be an indirect modulator of HIF-1α, since the function of prolyl hydroxylase domain-containing proteins (PHDs) is affected by ROS levels, which are the enzymes that sense oxygen tension and hydroxylate proline residues in HIF-1α and target it for proteasomal degradation [196]. In addition, HIF-1α induces small Mafs transcription through direct binding [128], providing continuous angiogenic signaling for tumors. Hence, c-Maf and small Mafs/Nrf2 regulate multiple steps of angiogenesis, including hypoxia stress, HIF-1α stability, VEGF expression level, and activation of vascular endothelial cells. By suppressing the temporal and spatial expression of these Mafs, we may be able to control the formation of the vascular network in cancer.

In summary, Mafs generally function as oncoproteins in most types of cancer due to their roles in promoting proliferation, metastasis, interaction between tumor and stroma, angiogenesis, and inhibiting apoptosis. However, c-Maf and MafF have dual effects on cancer, which may depend on varying upstream regulations and their diversity of function. Moreover, only a few studies exist regarding the role of NRL in cancer. Despite a few steps that have been taken in exploring the function and mechanism of NRL in cancer proliferation and apoptosis, more research is necessary to fully understand the link between NRL and cancer.

## The clinical applications and limitations of Mafs

### Mafs as a biomarker in cancer?

The lack of early symptoms in some types of cancers makes it difficult to diagnose cancer patients until an advanced stage [197–199]. Therefore, it is necessary to identify specific and sensitive biomarkers that can be used to effectively detect cancers at early stage and select cancer patients for individualized therapy. A number of recent studies have demonstrated that Mafs were key regulators in the pathogenesis of different types of malignant tumors [12, 107, 127]. In addition, Mafs show differential expression in a large number of human cancer tissues [56, 200, 201], offering hope for their latent use as indicators in predicting the diagnosis and prognosis of diverse cancers. For example, by analyzing the c-Maf expression level of tumor specimens from 123 multiple myeloma patients, c-Maf was identified as an independent unfavorable prognostic factor for overall survival [202]. Detection of MAFB represents a promising prognostic biomarker that stratifies a subset of patients with the shortest overall survival in osteosarcoma and HCC [120, 127]. MafF is also recognized as a potential biomarker in HCC and thyroid papillary carcinoma patients [127, 203]. On the contrary, the enhanced expression of MafF works as a better prognostic indicator in bladder cancer [204]. Moreover, enforced MafK expression is related to poor prognosis in triple-negative breast cancer patients [126]. Nevertheless, in developing Mafs as biomarkers for the diagnosis and prognosis of different types of cancers, several challenges need to be overcome. Firstly, it is not entirely clear how the expression levels of Mafs correlate with the progression of tumors, which needs to be further clarified. Secondly, the technical limitations of Mafs expression analysis and intra-tumoral heterogeneity may cause the discrepancies in the reliable biomarker role of Mafs. The last but not the least, the latent values of Mafs as biomarkers for cancer have only been confirmed in studies with modest sample sizes. Therefore, more convenient and accurate technologies should be explored to individually normalize and analyze Mafs expression. It is also crucial to deeply understand the relationships between Mafs expression and molecular tumoral heterogeneity. The utility of Mafs in stratification of patients into appropriate risk categories requires confirmation through more large-scale population-based studies.

### Mafs as a therapeutic target in cancer?

**Fig. 4:**
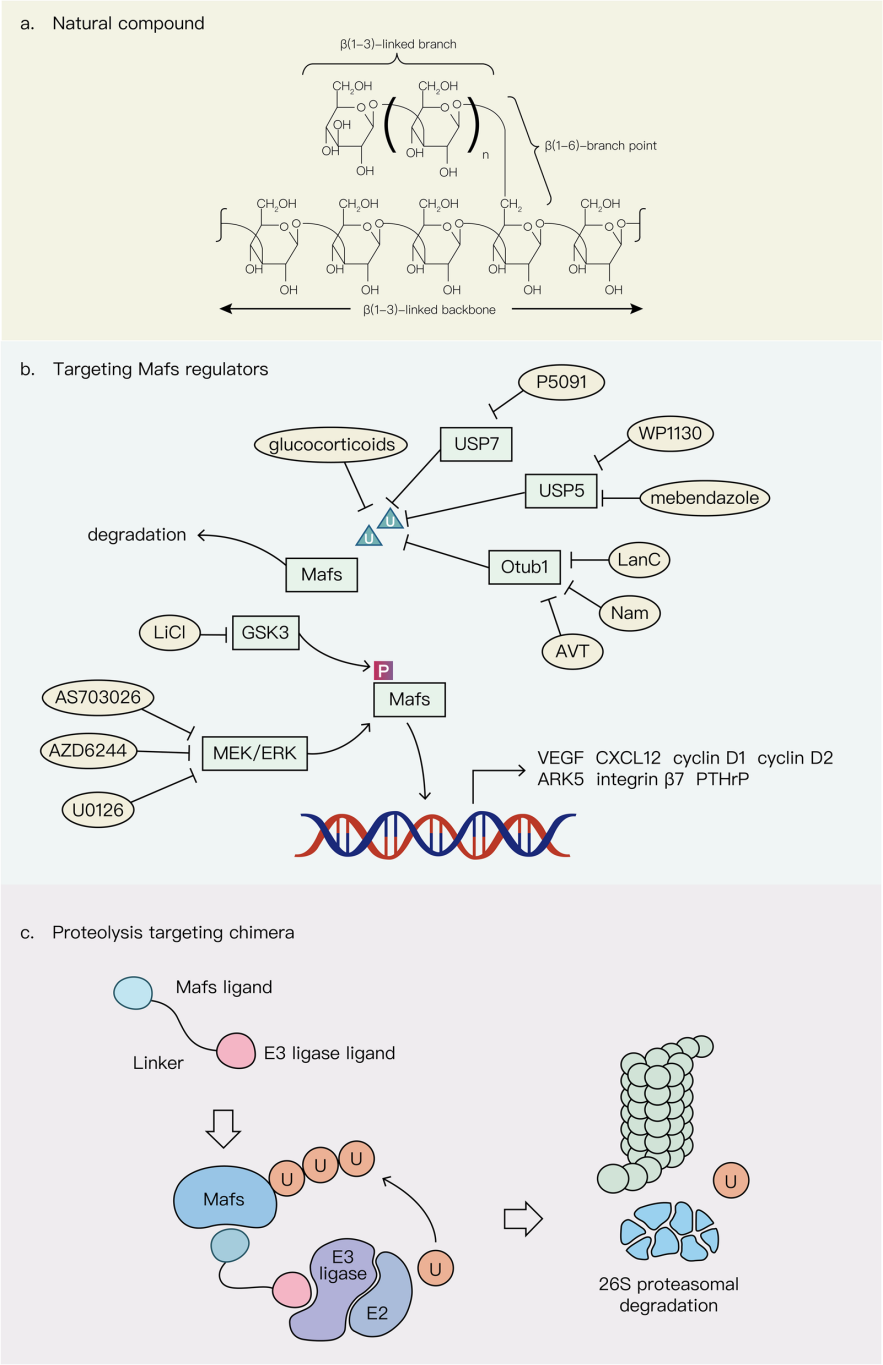
Potential strategies for Mafs targeting in cancers. **a** The structure of natural compound β-glucans targeting c-Maf. **b** Compounds targeting the upstream regulators of Mafs. **c** Proteolysis targeting chimera (PROTAC) for Mafs degradation

Enhanced expression of Mafs contributes to the proliferation, metastasis, tumor/stroma interaction and angiogenesis of different types of cancer [79, 116, 119, 121]. Some studies have revealed that Mafs overexpression conferred resistance to chemotherapeutic drugs such as bortezomib and cisplatin in various cancers [72, 143, 205]. Therefore, Mafs can act as efficient and potential therapeutic targets for cancer. Potential strategies including targeting Mafs regulators such as enzymes involved in posttranslational modifications and several Mafs-related signaling pathways as well as utilization of natural compound and PROTAC technology, can be applied to manipulate the expression of Mafs for cancer therapy (Fig. 4). Since MEK can upregulate c-Maf expression through the FOS transcription factor, targeting the MEK/ERK pathway by treatment with two typical MEK inhibitors U0126 and AZD6244 and a novel MEK inhibitor AS703026 inhibits MM proliferation and induces MM cell apoptosis [62, 206]. Given that Mafs are deregulated by posttranslational modifications, the regulation of Mafs stability is also a possible therapeutic approach. For instance, MafA, MafB and c-Maf are phosphorylated by the Ser/Thr kinase GSK3 in human MM cell lines. Lithium chloride (LiCl), a GSK3 inhibitor, targets these phosphorylation sites and specifically decreases the malignancy of Maf-expressing MM cell lines [207]. A subgroup of patients with MM who appear to have overexpressed large Mafs might benefit from lithium chloride treatment, which is already used to treat diabetes and neurodegenerative disorders in humans [208]. Similarly, glucocorticoids and P5091-mediated ubiquitylation and degradation of c-Maf specifically inhibit the proliferation rate of MM cell lines that overexpress c-Maf and decrease the expression of c-Maf transcriptional targets including integrin β7 and cyclin D2 [11, 209]. However, these chemicals for cancer therapy have only been confirmed in vitro, and further animal experiments and clinical trials are necessary for a thorough understanding of their therapeutic effects. In addition to Mafs regulator related drugs, β-glucans, a newly discovered natural compound targeting c-Maf, delays tumor growth by transforming M2-like macrophages into an M1-like phenotype that corresponds with downregulated c-Maf levels in non–small cell lung cancer. A clinical trial of β-glucans was initiated in NSCLC patients. Although no information on outcome was yet available, a notable reduction in c-Maf mRNA expression was identified in circulating CD14dimCD16 + myeloid cells [210, 211]. Since small molecule inhibitors targeting Mafs are lacking, screening or synthesizing more specific chemotherapeutic drugs that target Mafs are able to offer a novel and useful therapeutic strategy for cancer therapy in the future. Furthermore, the absence of specific inhibitors that target Maf transcription factors may be due to the lack of structurally stable small molecule binding pockets and metastable regulatory sites as well as clear regulatory mechanisms, making Mafs undruggable proteins [212]. Proteolysis targeting chimeras (PROTACs) are used to degrade targeted proteins through the ubiquitin–proteasome system, a natural intracellular protein degradation system. The action of PROTACs is not dependent on ligand binding pockets. Molecular glue, an important component of PROTAC, can target any region of the protein to perform its function [213]. Hence, the application of PROTACs in Mafs degradation is full of opportunities in the treatment of diverse tumors.

Although there are potential strategies for developing Mafs-targeting drugs for multiple cancers, targeting Mafs selectively and efficiently is still challenging. One of the reasons is the insufficiency of structural information of Mafs and their CNC partners. It is crucial to acquire the three-dimensional structure on Mafs and identify proper ligand-binding sites for the design and discovery of optimal drugs directly targeting Mafs. Another challenge is the lack of identifying molecular glues that are specific to a certain tissue or a tumor type in the Mafs-targeting use of PROTACs. More specific molecular glue will be beneficial to reduce the on-target toxicity in the clinic. Finally, a more comprehensive understanding of the physiological functions of Mafs and their relationships with a specific disease is still critical for the development of Mafs-targeted treatment. Due to the complexity of diverse signaling pathways converging at Mafs, the context of potential crosstalk between pathways must be taken into account in exploring an efficient strategy to directly or indirectly target Mafs.

## Conclusion and Future perspectives

Mafs used to be rendered key modulators only in multiple physiological processes including embryonic development, tissue formation and insulin secretion. In recent years, structural and molecular biology as well as bioinformatics analyses have provided valuable insights into their critical regulatory roles in tumorigenesis as they govern the expression of many genes involved in cell proliferation, apoptosis, angiogenesis, tumor/stroma interactions and drug resistance. The binding affinity of Mafs as well as the expression and activity of target genes that are precisely controlled by Mafs become disordered during cancer development. Individual proteins or cofactors interacting with selected Mafs have also been identified in conjunction with several Mafs target genes. These advances in Mafs studies have occurred due to the recent technological advances such as chromatin immunoprecipitation technology, so that we can move forward to resolve some significant questions and explain many puzzles. The application of chromatin immunoprecipitation combined with sequencing not only enables us to identify the DNA target sites of Mafs on a global scale but also assists in revealing how promoter discrimination can be achieved despite the stereotypic binding preference to the MARE. However, there are some defects in the use of chromatin immunoprecipitation. It is difficult to capture and purify dynamic Mafs-related transcriptional complexes at promoter regions following different signaling pathways and changeable microenvironments in vivo. Furthermore, some nonspecific proteins might be detected due to the crosslinking of formaldehyde. Therefore, we can foresee that the improvement of existing methods and explorations of new technology can offer the biochemical basis for disentangling the intricate Mafs-dependent crosstalk at promoter sites and help to develop feasible Mafs-based strategies in cancer therapy.

## Data Availability

Not applicable.

## Abbreviations

3’UTR: 3’-Untranslated region
AKT: AKT serine/threonine kinase
AP-1: Activated protein 1
ARK5: AMPK-related protein kinase 5
ARE: Antioxidant response element
BACH: BTB domain and CNC homolog
bZIP: Basic leucine zipper
CCA: Cholangiocarcinoma
CD13/APN: Alanyl aminopeptidase, membrane
CDK: Cyclin-dependent kinase
CHD8: Chromodomain helicase DNA-binding protein 8
CNC: Cap-n-Collar
CREs: CAMP responsive elements
Csf-1r: Colony-stimulating factor 1 receptor
CXCL12: C-X-C motif chemokine ligand 12
circRNAs: Circular RNAs
c-Myc: MYC proto-oncogene
DLBCL: Diffuse large B-cell lymphoma
DNMT3B: DNA methyltransferase 3B
ECM: Extracellular matrix
EMT: Epithelial to mesenchymal transition
ERK: Mitogen-activated protein kinase
FoxA2: Forkhead box A2
GPNMB: Transmembrane glycoprotein non-metastatic B
GSK3: Glycogen synthase kinase 3
HBZ: HTLV-1 basic leucine-zipper factor
HCC: Hepatocellular carcinoma
HER: Extended homology region;
HERC4: HECT and RLD domain containing E3 ubiquitin protein ligase 4
Hox: Homeobox protein
IL11: Interleukin 11
LiCl: Lithium chloride
lncRNAs: Long non-coding RNAs
Mafs: Maf proteins
MARE: Mafs recognition element
MATα1: Methionine adenosyl transferase 1A
MEK: Mitogen-activated protein kinase
MENIN: MEN1 protein
MM: Multiple myeloma
MMP: Matrix metalloproteinase
MyoD: Myogenic differentiation 1
MPNST: Malignant peripheral nerve sheath tumor
miRNAs: MicroRNAs
ncRNAs: Noncoding RNAs
Nkx2.2: NK2 homeobox 2
Nrf2: NFE2 like bZIP transcription factor 2
NRL: Neural retina-specific leucine zipper
NSCLC: Non–small cell lung cancer
NQO1: Quinone oxidoreductase-1
Otub1: OTU deubiquitinase, ubiquitin aldehyde binding 1
Pax6: Paired box 6
PDGF: Platelet-derived growth factor
Pdx1: Pancreatic and duodenal homeobox 1
PHDs: Prolyl hydroxylase domain-containing proteins
PROTAC: Proteolysis targeting chimera
PTHrP: Parathyroid hormone like hormone
PTMs: Post-translational modifications
RISC: RNA-induced silencing complex
STAT3: Signal transducer and activator of transcription 3
TAMs: Tumor-associated macrophages
TICs: Liver tumor-initiating cells
TREs: TPA responsive elements
UBE2O: Ubiquitin conjugating enzyme E2 O
USP5: Ubiquitin specific peptidase 5
VEGF: Vascular endothelial growth factor
